# Limb salvage after gas gangrene: a case report and review of the literature

**DOI:** 10.1186/1749-7922-6-28

**Published:** 2011-08-17

**Authors:** John Aggelidakis, Konstantinos Lasithiotakis, Anastasia Topalidou, John Koutroumpas, Georgios Kouvidis, Paulos Katonis

**Affiliations:** 1 Department of Orthopaedic Traumatology, University Hospital of Heraklion, Voutes, Heraklion, 71100, Greece; 2 Department of General Surgery, University Hospital of Heraklion, Voutes, Heraklion, 71100, Greece; 3 Alexander S. Onassis Public Benefit Foundation, Voutes, Heraklion, 71100, Greece

## Abstract

Gas gangrene is a necrotic infection of soft tissue associated with high mortality, often necessitating amputation in order to control the infection. Herein we present a case of gas gangrene of the arm in an intravenous drug user with a history of intramuscular injections with normal saline in the shoulder used to provoke pain for recovery after drug induced coma. The patient was early treated with surgery and antibiotics rendering possible the preservation of the limb and some of its function. Additionally, a review of the literature regarding case reports of limb salvage after gas gangrene is presented.

## Background

Gas gangrene or Clostridial myonecrosis is a necrotic infection of skin and soft tissue and it is characterized by the presence of gas under the skin which is produced by Clostridium. It is a potentially lethal disease which spreads quickly in soft tissues of the body. Tissue necrosis is due to production of exotoxins by spore forming gas producing bacteria in an environment of low oxygen. Gas gangrene is subclassified in two categories. Traumatic or postoperative is the most common form accounting for 70% of the cases followed by spontaneous or non traumatic gangrene. *C. perfringens *is isolated in approximately 80% of patients presenting with traumatic gas gangrene followed by *C.septicum, C.novyi, C.histolyticum, C.bifermentans, C.tertium *and *C.fallax *[1-3]. Herein we report a case of gas gangrene which was treated early with surgical debridement and enabled salvage of the limb with significant preservation of its function. Additionally, a review of the literature regarding cases of limb salvage after gas gangrene is presented.

## Case Presentation

**Figure 1:**
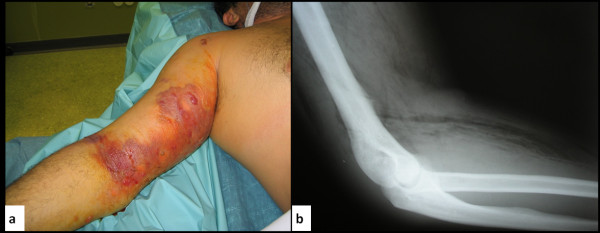
**Gas gangrene in an illicit drug user.  **a. One and half hours after his admission in the emergency department. b. X-ray of the affected limb revealing gas in soft tissues.

A 35-year-old Caucasian man with a history of chronic intravenous drug use presented to the emergency department with right upper limb pain and swelling lasting 24 hours. His initial vital signs were notable for temperature of 39°C, respiratory rate of 25 breaths per minute, heart rate of 120 beat per minute and blood pressure of 141/76 mmHg. He was distressed and on clinical examination severe edema of the upper limb, erythema, blistering of the arm and crepitus over the shoulder and arm was noted [Figure 1a]. At this time, motor and sensory function of the limb was not impaired and pulses of the radial and ulna artery could be palpated. His past medical history consisted of a diagnosis of hepatitis C. Intramuscular injections with normal saline in the shoulder were also reported. This is a practice among illicit drug users used to provoke pain for recovery after drug induced coma.

Blood counts showed a white blood cell count of 10.7 K/μL (normal range 3.5-10.0 K/μL) (88.6% neutrophils, 6.9%lymphocytes, 0.1%monocytes), hemoglobulin 13.6 g/dl (normal range 14-18 g/dl), platelet count 161 K/μL (normal range 150-450 K/μL). His creatinine phosphokinase was elevated at 3594 IU/L (normal range 40-148 U/L), c-reactive protein was elevated at 7.29 mg/dl (normal range < 1 mg/dl) and SGOT/SGPT were two times above higher normal limits. His electrolytes and coagulation profile were within normal limits.

**Figure 2:**
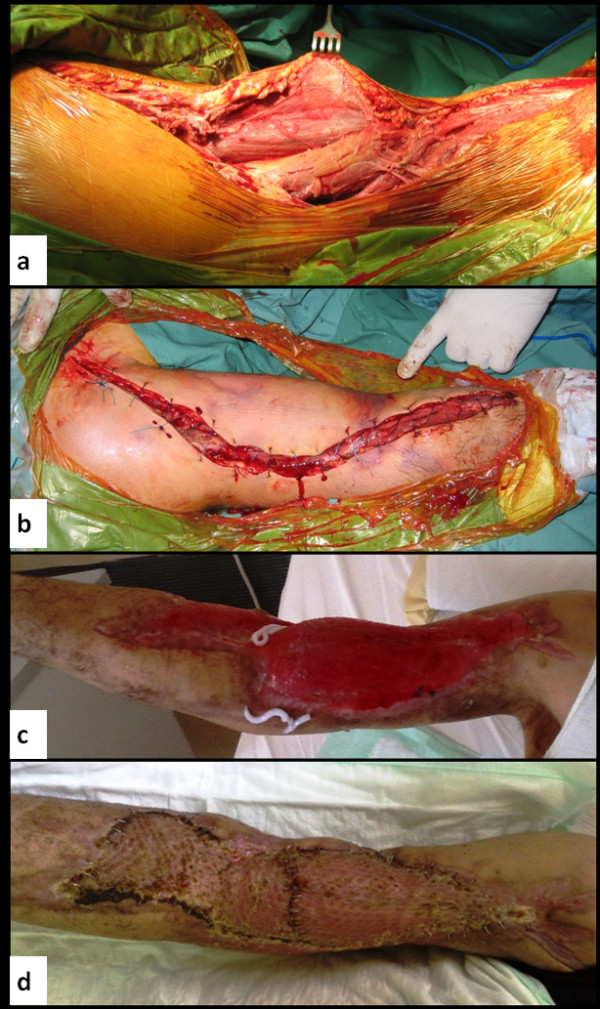
**Surgical treatment of gas gangrene with preservation of the affected limb.**   a. Intraoperative figure showing necrosis of significant proportions of biceps brachii and the flexors of the forearm. b. Approximating sutures after broad resection of necrotic tissues of arm and forearm. c. Postoperative day 50: Healing with granulation of the tissue. d. Four months postoperatively: Restoration of skin deficits with the use of free skin flaps.

An X-ray of the affected limb revealed gas in soft tissues suggestive of gas gangrene [Figure 1b]. Empirical broad spectrum antibiotic treatment was immediately initiated consisting of piperacillin/tazobactam, clindamycin and vancomycin in usual dosages. Within one hour swelling of soft tissues was expanded to the forearm and neck medially [Figure 2a]. The general condition of the patient was worsening with severe pain and hoarseness and he was intubated due to threatened airway. Within two hours since his admission, the patient was guided to the operating theater and underwent arm and forearm fasciotomy due to threatening compartment syndrome and broad surgical debridement and drainage of the infected areas. A Henry type anterior shoulder incision was used from the anterior deltoid muscle to the forearm with division of the transverse carpal ligament.

Extended subcutaneous emphysema was noted, with foul smelling areas of necrosis in most of biceps brachii and the flexors of the forearm. Broad resection of necrotic tissues of arm and forearm was done. Thorough mechanical irrigation of the affected area was performed using normal saline, hypertonic solutions and the Stryker irrigation-suction device. Approximating tension sutures were used and the wound was let to be healed by third intention [Figure 2b]. Subsequently the patient was transferred to the intensive care unit. Cultures of tissue specimens obtained intraoperatively revealed Staphylococcus epidermidis, Clostridium perfringens and Staphylococcus aureus.

Postoperatively the patient remained in the intensive care unit intubated and in septic shock. The first postoperative day he developed acute renal failure attributed to myoglobinuria requiring hemodialysis. The second postoperative day his platelet count was decreased to 45Κ/μL and increased gradually the following days. According to the results of antibiogram meropenem 1 gr 12 hourly was administered the 3^rd ^postoperative day. Daily surgical debridement with resection of additional necrotic tissue was performed in the intensive care unit. His temperature returned to normal on postoperative day 10 and his general condition was gradually improved thereafter. He was discharged from the intensive care unit on postoperative day 30. In the orthopedic ward he remained afebrile and his wound was progressively healing with granulation of the tissue and regression of the foci of necrotic infection [Figure 2c]. Blood supply of the limb was adequate. However, significant motor and sensor neural deficits of the radial and ulnar nerve were noted. Limb physiotherapy was administered on daily basis. Four months postoperatively, skin deficits were restored with the use of free skin grafts from the femoral region [Figure 2d]. At this time flexure and extension of the elbow and shoulder against gravity was possible along with minimal active movement of the wrist and fingers.

## Review of cases reported in the literature

This review included Medline reported adult cases of limb salvage following gas gangrene (clostridial myonecrosis) until June 2011. Only articles in the English language, with reported culture results, in which limb salvage was attempted and the outcome of that attempt was clearly indicated were included. Data extracted from each article included age, gender, relevant and general history, previous diagnoses, infection location, clinical presentation, antimicrobial treatment, surgical treatment, complications of the infection, duration of hospitalization and functional outcome.

We identified eleven cases which are presented in Table 1. There were two cases of multimicrobial myonecrosis (clostridia in combination with Gram positive cocci). Males dominated in this sample consisting 90% of total. Conditions related with clostridial myonecrosis could be broadly classified as posttraumatic (n = 3, postoperative, after injury or intravenous use of illicit drugs) and related with gastrointestinal disease (n = 6, colon cancer, chronic pancreatitis). Gastrointestinal disease, especially colon cancer, was invariably associated with *C. septicum *infection. Diabetes mellitus was present in three cases. Lower limb, particularly thigh was the most common anatomical site of the infection. In most of the cases the duration of symptoms before admission did not exceed two days. One patient reported by Kershaw et al [4] experienced pain lasting 6 days prior to admission which is considerable higher compared with the rest of the patients. Clinical presentation involved pain localized in the affected limb (90%), fever (70%) and crepitus (45%). Other presenting symptoms included swelling, discoloration, induration of the affected limb, tenderness, stiffness of involved joints, abdominal pain, nausea and vomiting.

**Table 1:** Cases of limb preservation after treatment of gas gangrene (Clostridial myonecrosis)

Age/Gender/Reference	Comorbidity, Previous history	Localization/Cultures results	Clinical presentation	Antibiotics/Other treatment	Complications/Hospitalization (days)/Functional status
35/M [Present case]	Intravenous drug user, Hepatitis C	Shoulder/C. perfrigens, Staph aureus, Staph epidermidis	Pain, fever, swelling, crepitus	Pip/Taz, Clind, Vanc → Meropenem/Skin grafting	Septic shock, myoglo-binuria, RF/120d/limited function

54/M [10]	DM, cecal cancer	Arm/C septicum	24 hr arm/abdominal pain, fever, nausea, vomiting, diarrhea, shoulder tenderness, induration, crepitus	Pip/Taz, Clind, Vanc	Anemia/NR/NR

37/M [5]	Posttraumatic Head fracture	Shoulder/C perfrigens S epidermidis	shoulder pain, fever, agitation, crepitus	Vanc → Pen/Clind/Metr → Cefo, Metro → Pen/Metro → Metro p.os	Anosmia/40d/Normal

26/M [9]	Intravenous drug user	Lower limb/C perfringens, Beta- Streptococci, enterococci	Suspected DVT, thigh/left iliac fossa tenderness	Pen, Clind, Metr/femoral artery vascular grafting	Femoral vein, artery and nerve erosion/126d/Mobile

49/M [23]	Postoperative	Hand/C perfrigens C sordellii	1^st ^postoperative day pain/fever	Pen	-/21d/normal

55/M [12]	DM, peripheral vascular disease, cecal mass	Hip/C septicum	Pain, fever, crepitus	Pip/Taz, Clind, Ceft→Pip/Taz, Clind	RF, myoglobinuria/NR/NR

58/M [6]	Posttraumatic	Heel/	Foot pain, fever,	Antibiotics, hyperbaric oxygen, Skin grafting	MOFS/78d/normal

32/M [11]	Postoperative	Lower limb/C septicum	Pain, crepitus	NR	NR/NR/NR

83/M [14]	Sciatica, pneumonia, colon cancer	Hip, thigh/C septicum	3 days, hip pain, fever, nausea, vomiting	Vanc, Genta, Imip/Sil → Am/Cl/Right hemicolectomy	-/16d/ambulated with assistance

47/M [4]	chronic pancreatitis, DM, pentazocin injection sites.	Thigh - buttock/C perfrigens	6 day pain, swelling, fever,	Pen, Metr, polyvalent clostridial antitoxin,/Skin grafting	Respiratory failure,/NR/normal

25/M [7]	Crush injury	Leg/C perfrigens	Pain, fever, limb discoloration, edema, crepitus	Cefalotin → Pen, hyperbaric oxygen/skin-bone grafting	-/180d/able to bare weight

48/F [24]	Posttraumatic	Knee/C perfrigens	Pain, stiffness, tenderness	Terra → Pen, Gas gangrene serum	-/21d/normal

Pip/Taz: piperacillin/tazobactam, Clind: clindamycin, Vanc: vancomycin, Pen: penicillin G, Metr: metronidazole, Genta: gentamycin, Imip: imipenem, Sil: silastatin, Am/Clav: amoxicillin/clavulate, Terra: terramycin,

DM: diabetes mellitus,

UC: ulcerative colitis,

DVT: deep venous thrombosis

MOFS: multiorgan failure

RF: renal failure

NR: not reported.

All patients underwent wide surgical debridement of the affected area and were administered antimicrobial treatment. Three out of eleven patients underwent at least a second wound debridement after initial operation [5-7]. A detailed list of antimicrobial regimens used in these patients is presented in Table 1. Penicillins, clindamycin or metronidazole were included in the initial antibiotic regimen in 70% of cases. Other common antimicrobial agents used were vancomycin, gentamycin, imipenem and cefalosporins. Adjunctive therapy with hyperbaric oxygen was administered in two patients. In one patient a polyvalent clostridial antitoxin was administered [4]. However, to our knowledge no commercially available polyvalent clostridial antitoxin exists in Europe and in the US. Skin grafting to cover affected areas was required in three cases. Surgical complications included a case of erosion of the femoral artery treated with vascular grafting, severe bleeding of the groin area that was managed with ligation of profunda femoris artery and its branches. The most serious systemic complications of the infection were respiratory failure, renal failure, sepsis and resultant multiorgan failure. Notably, one patient who developed respiratory failure was receiving intramuscular pentazocin, an opioid analgesic for chronic pancreatitis associated pain. Pentazocin is not indicated for patients with pancreatitis and can itself depress critically the respiratory function [4,8]. Hospitalization ranged variably between 16 and 126 days and was relatively longer in patients with serious systemic complications of the disease. Functional status of the salvaged limb was reported in eight cases, five of them regaining normal function of the affected limb.

## Discussion

Gas gangrene of the limbs is a rare infection due to anaerobe bacteria associated with high morbidity and mortality. Amputation is usually necessary to control infection and save life whereas functional limb preservation is rare [1]. Intravenous drug users are considered at high risk for gas gangrene and it has been shown that Clostridia are able to survive in heroin preparations being mixed with citric acid and heated [2]. Moreover, repeating trauma of soft tissue resulting from peculiar practices among illicit drug users, as the intramuscular injections with normal saline in our case, introduce organisms directly into deep tissue and create an anaerobic environment that is ideal for the proliferation of Clostridia. Such anaerobic environment also results from crash type injury, contaminated open fractures and retained foreign material and is associated with *C.perfrigens *gas gangrene [3,5,7,9]. Spontaneous gas gangrene of the limbs is due to *C. septicum *in the vast majority of cases. *C. septicum *translocates from the gut suffering from a benign or malignant disease and causes metastatic infection [1,10-12].

Incubation time is short usually less than 24 hours and the physical finding of crepitus is characteristic finding in the setting of soft tissue infection [5,7,10-12]. The sudden onset of pain, rapidly progressive soft tissue infection, development of blisters containing foul smelling brownish liquid with gas bubbles, soft tissue induration and discoloration may also be present [7,10]. Plan X-rays identify gas in deep tissues and CT or MRI may assess spreading of infection along fascial planes. Bacteremia occurs in approximately 15% of patients and normally develops several hours before skin manifestations in the case of spontaneous gangrene [1]. Needle aspiration or biopsy may provide etiological agent but no diagnostic test or hyperbaric oxygen therapy should replace or delay surgical and antimicrobial treatment.

Signs of systemic toxicity develop rapidly and many patients present with septic shock at the time of their admission to the hospital [13]. However, the cases of limb salvage reported in the literature did not present with fulminant systemic disease and only four out of eleven, including our patient developed serious complications due to their disease (Table 1). This may indicate a less aggressive form of the disease or a better treatment outcome because of early diagnosis. Liver necrosis, jaundice, hemolytic anemia and renal failure are some serious systemic complications of clostridial myonecrosis. Renal failure is attributed to the effects of hypotension, myoglobinuria, hemoglobinuria and direct nephrotoxicity of clostridial toxins [1]. Severe pain, toxicity and high creatinine phosphokinase levels with or without radiographic findings are indications for surgery in order to achieve early debridement and obtain tissue for appropriate cultures.

The mainstay of treatment is early aggressive surgical intervention, antibiotic therapy and intensive care support. Delay of the operation for more than twelve hours is associated with higher overall morbidity [13]. Cases of limb salvage after gas gangrene reviewed in this article were almost invariably operated immediately after their admission with the diagnosis of gas gangrene and with symptoms of duration of less than 48 hours. In only two cases diagnosis of gas gangrene was delayed for more two days even though the patients had been previously examined by their doctors [4,14].

Wide resection of all necrotic tissue is necessary. Only viable muscle that bleeds when cut or contracts upon stimulation with electrodiathermy should be left behind. Fasciotomies are necessary to prevent compartment syndrome. Evidence based indication for amputation of limbs affected with gas gangrene does not exist. Unlike several scoring systems existing for assessing the need for amputation in traumatic limb injury (Lange's, the predictive salvage index, the limb score injury, the limb salvage index, the mangled extremity syndrome index and the mangle extremity severity score) no scoring system has been developed for necrotic infections of the limbs. Even though some of the components of the aforementioned scoring systems may also be applied in limb gangrene, they have not been validated and essentially they cannot replace experience and good clinical judgment [15].

With improvements in prehospital care, acute resuscitation and surgical techniques, surgeons more often are faced with situations in which a severely compromised limb can be preserved although this involves substantial compromises. Realistic likelihood of functional recovery of the limb must be balanced against the risk of death associated with attempts to preserve a limb. Amputation might be beneficial in cases where no residual function of the limb is expected postoperatively. This implies major deficit of its neurovascular supply. Major nerve involvement may lead to preservation of a useless extremity that is worse than no limb at all [15].

For the lower limb, destruction of the tibial nerve is considered an indication for below-knee amputation since the functional result of the preservation of the limb is worse compared with the use of prosthesis. Modern prosthetics often provide better function than many "successfully salvaged" limbs. For the upper limb, even minimal preservation of the movement and sensation might be beneficial for the patient (handle a wheel chair, use computer systems etc) and generally provides better function compared with prosthesis. Non palpable pulse of the radial or dorsalis pedis artery intraoperatively should lead to sonographic assessment of the vascular supply of the limb. If no venous return is seen on triplex, amputation should be strongly considered. Severe, irreparable vascular injury in an ischemic limb is another indication for amputation. Before performing an amputation, a vascular surgery consultation should be considered if available without delaying the treatment decision [15,16]. Improved techniques currently allow for revascularization of limbs that previously would have been unsalvageable. Revascularization is not without risk, however [9,15]. Attempts to salvage a severely compromised limb may lead to metabolic overload and secondary organ failure. Comorbid medical conditions must also be considered before heading down a long road of multiple operations to save a limb [15].

Even though cases with aggressive infection presenting with systemic complications due to gas gangrene of the limb are more likely to have more advanced local infection which precludes limb salvage, there is no evidence that amputation controls infection better than adequate wide surgical debridement. Therefore, in our patient the treatment decision for limb salvage was not influenced by the presence of systemic complications. It was rather based on the estimation of what is left behind after an adequate resection of all devitalized tissue. If limb salvage is attempted, one must take into account that postoperative daily surgical exploration might be necessary for several days until all necrotic tissue is removed. In cases of limb salvage after gas gangrene reported in the literature, serial debridement following initial surgery was necessary only in four patients including our case. This might indicate a more adequate initial operation in cases with limb preservation or a less aggressive form of disease in these patients [5-7].

Even though gas gangrene of the limb is an extremely emergency surgical condition individual patients' preferences after thorough information should be taken into account. First a decision is taken whether the limb can be saved. If the limb can be preserved the decision whether it should be saved should come in concert with the patient. The tradeoffs involved with protracted treatment course of limb salvage versus immediate amputation and prosthetic fitting should be made clear to the patient. Saving the limb, often comes at a great cost. Multiple operations to obtain bony reunion and soft tissue coverage are often necessary. Chronic pain and drug addiction also are common problems of limb salvage because patients endure multiple hospital admissions and surgery, isolation from their family and friends, and unemployment [15,16]. In the end, despite heroic efforts the limb ultimately could require an amputation or a "successfully salvaged limb may be chronically painful or functionless [17,18]. The worst case scenario occurs when a limb must be amputated after the patient has endured multiple operations of an unsuccessful salvage or after years of pain following a "successful" salvage [18]. On the other hand, early amputation and prosthetic fitting has been shown to be associated with decreased morbidity, fewer operations, shorter hospital course, decreased hospital costs, shorter rehabilitation in cases of traumatic limb injury [15]. Thus, it is important to present all information from the very beginning so that the patient is able to make educated decisions regarding which course to follow. The subjective importance of body image for the patient, the possibility of prolonged hospitalization, financial burden and possible social isolation should be discussed with the patient in order to help them make real informed decisions [15,16].

Prompt initiation of antimicrobial treatment covering aerobic and anaerobic organism is critical. In fact, early antimicrobial treatment was initiated in all cases with preservation of the limb after operation for gas gangrene. Initial empirical antibiotic treatment should cover Clostridia, Gram positive cocci aerobes and anaerobes. The optimal combinations of antibiotics as well as the duration of the treatment have not been defined in appropriate clinical trials so far. Ampicillin-sulbactam or piperacillin-tazobactam or ticarcillin-clavulate in combination with clindamycin or metronidazone are suggested empiric regimens, whereas antibiotic treatment should be tailored according to the susceptibility results [1,19]. Specific treatment for post traumatic gas gangrene due to *C. perfrigens *should consist of Penicillin (3-4MIU every 4 hours i.v.) plus Clindamycin (600-900 mg every 8 hours i.v.). In cases of spontaneous gas gangrene due to *C. septicum *antimicrobial treatment should include vancomycin (1 g every 12 hours i.v.) or metronidazole (500 mg every 8 hours i.v.) because this species may be resistant to penicillin or clindamycin [19]. Interestingly, in the vast majority of cases with limb salvage after gas gangrene reviewed here, empiric as well as specific antibiotic regimen was in concordance with the aforementioned suggestions.

Neutralization of clostridial or streptococcal circulating toxins by the use of intravenous immune globulin has shown promising results but there are no data to support a strong recommendation for its regular use in patients with gas gangrene [20]. Adjunctive hyperbaric oxygen therapy has been suggested for patients with aggressive soft tissue infections and has been shown to increase survival in animal model and in humans but no prospective controlled trials have been contacted in humans so far. Better definition of necrotic tissue facilitating more precise debridement and its bacteriostatic effects on clostridia both in vivo and in vitro is the rationale for the use of hyperbaric oxygen therapy in patients with gas gangrene [21,22].

In most of the patients with limb preservation after gas gangrene, a residual function of the affected limb was present. In half of them functionality of the limb was characterized as normal. Patients with limited function of the preserved limb had generally longer duration of hospitalization. This might be at least in part because these patients, as our case, needed several interventions following initial surgery until the limb re-attained as much as possible of its functionality. This prolongation of hospital stay is well balanced by the invaluable benefit of functional limb salvage. Whether the preservation of the limb makes postoperative recovery more severe is essentially the question whether amputation offers better control of the infection compared with adequate debridement. Again there is no evidence that amputation controls better the infection compared with adequate debridement. However, it is plausible that amputation may achieve margins that are wider and clearer of infection if it is compared with an inadequate debridement in order to "save" the limb [15,16].

In conclusion, physician and emergency medicine personnel should always maintain high index of suspicion for necrotizing infections in illicit drug users presenting with soft tissue infections. Early surgical debridement, antimicrobial treatment and intensive care monitoring may lead to survival with limb salvage in carefully selected patients.

## Consent

Written informed consent was obtained from the patient for publication of this case report and accompanying images. A copy of the written consent is available for review by the Editor-in-Chief of this journal.

## List of abbreviations

CT: computerized tomography; MRI: magnetic resonance imaging; SGOT/SGPT: serum glutamic oxaloacetic transaminase/serum glutamic pyruvic transaminase.

## Competing interests

The authors declare that they have no competing interests.

## Authors' contributions

IA and AT had the original idea and drafted the manuscript. PK and KL drafted, reviewed, finalized and revised the manuscript. GK and JK searched the literature and prepared the figures. All authors read and approved the final manuscript.
